# An Antivaccination Crusade

**Published:** 1910-02

**Authors:** 


A Monthly Review of Medicine and Surgery.

EDITOR

WILLIAM WARREN POTTER, M. D.

All communications, whether of a literary or business nature, books for review and xchanges, should be addressed to the editor, 238 Delaware Avenue, Buffalo, N.Y.

Vol. Lxv. FEBRUARY, 1910. No. 7

### An Antivaccination Crusade

WERE it not for the serious consequences liable to attend such a movement, one might be tempted to write jokefully of the attitude of the citizens of North Tonawanda, or some of them, toward the vaccination problem. The opportunity presents, in stage setting and characters, material for an opera bouffe that might rival any of Gilbert and Sullivan's productions. The Poobah is there, and so is the lord high executioner,—whose other name is Smallpox,—and even the great Master of the Posts himself is ready to take part, and whose

> Object all sublime,
> He hopes to achieve in time,
> To fit the punishment to the crime,
> * * * the punishment to the crime.

The stage is made ready, the actors are about to enter, when lo! a messenger arrives from Washington who beckons the Master of the Posts aside. Nobody appears to have overheard their conversation, but it is believed the Washington man brought a message from the Generalissimo of the Posts, to the effect that Uncle Samuel is a believer in vaccination, requiring all his soldier boys to submit to the operation on enlistment, if not already protected; and further, that he would not permit the measure to be discredited by any of his subordinates inasmuch as it was purely a scientific question, only to be dealt with by the medical profession. Whereupon, the Master of the Posts turned over the chief command to the Grand High Burgomaster and retired from the stage.

The moral of all this Offenbachish setting is not far to seek. Jenner more than a century ago discovered a preventive measure against smallpox, known as vaccination, which arrested the spread of the disease and nearly stamped it out. It has served to fulfil its purposes ever since when the methods of its application are properly observed. The state decrees that school children must be vaccinated; this is so ordered by the advice of its public health officers and by that of the medical profession in general.

It is not wise, therefore, for the laity to oppose a scientific fact so well established as vaccination. It is against reason; it is against humanitarianism. If such a movement should be permitted to progress, if an organised league against vaccination should succeed in overthrowing established custom in regard to its employment, a sorry day for the people would ensue. We should next hear of organised opposition to the use of antitoxin in diphtheria, as well as against the various other serums now in accepted use or in the experimental stage.

We are not of those, however, who fear that the North Tonawanda league of antivaccinationists will pass beyond the opera bouffe stage. The comical is of short duration, while the serious lasts for all time. The contest is ever between superstition and ignorance on the one hand, and science and knowledge on the other.

